# Does Bilingualism Influence Cognitive Aging?

**DOI:** 10.1002/ana.24158

**Published:** 2014-06-02

**Authors:** Thomas H Bak, Jack J Nissan, Michael M Allerhand, Ian J Deary

**Affiliations:** 1 Department of Psychology, University of EdinburghEdinburgh, United Kingdom; 2 Centre for Cognitive Aging and Cognitive Epidemiology, University of EdinburghEdinburgh, United Kingdom

## Abstract

Recent evidence suggests a positive impact of bilingualism on cognition, including later onset of dementia. However, monolinguals and bilinguals might have different baseline cognitive ability. We present the first study examining the effect of bilingualism on later-life cognition controlling for childhood intelligence. We studied 853 participants, first tested in 1947 (age = 11 years), and retested in 2008–2010. Bilinguals performed significantly better than predicted from their baseline cognitive abilities, with strongest effects on general intelligence and reading. Our results suggest a positive effect of bilingualism on later-life cognition, including in those who acquired their second language in adulthood.

Recent studies suggest that bilingualism improves later-life cognition^1^ and delays the onset of dementia.^2^,^3^ The main limitation of this research lies in the bilingualism-associated confounding variables (eg, ethnic/environmental differences, immigration).^4^ Although a recent study succeeded in minimizing the environmental factors,^5^ another confound remains extremely difficult to tackle: reverse causality. Bilinguals might have different baseline characteristics from monolinguals; instead of bilingualism leading to cognitive differences, original differences (eg, childhood intelligence [CI]) could lead to bilingualism. This confound is particularly difficult to address, because it requires knowledge of prior levels of intelligence.

The Lothian Birth Cohort 1936 (LBC1936)^6^,^7^ offers an opportunity to overcome this confound. The participants took an intelligence test in 1947 at age 11 years, and were retested in 2008–2010. Reflecting the society of its time, the cohort is remarkably homogeneous; they are English native speakers, of European origin, born, raised, and living in and around Edinburgh. None was an immigrant. Thus, LBC1936 data allowed us to address, for the first time, the question whether learning a second language influences later cognitive performance after adjusting for CI. We predicted the strongest influence of bilingualism on frontal executive functions,^8^,^9^ additional benefits of multilingualism,^1^,^4^ and a better performance in bilinguals using both languages actively, although this variable has not been studied.

## Subjects and Methods

### Participants

LBC1936 Wave 1 testing included 1,091 participants of the Scottish Mental Survey 1947.^6^,^10^ Of those, 866 returned for the Wave 2 assessment in 2008–2010,^7^ and 853 (410 female, 443 male, age = 70.91–74.15 years, mean = 72.49, standard deviation = 0.71) completed the bilingualism questionnaire. Thirteen subjects, born abroad of British parents, moved to Scotland before the age of 11 years. The analysis conducted with and without these participants showed small differences and similar effect sizes, so we report the results from the full sample. A power analysis (G*Power 3.1.5^11^), with a bilingualism effect expressed as a partial *R*^2^ of 0.02 in a multiple regression model of 9 predictors, required a sample of 640 for a power of 0.95, deeming our sample sufficient.

### Assessment of Bilingualism

The participants were asked in a questionnaire whether they had learned any languages other than English (L2), how many, at what age, and how often they used them (daily/weekly/monthly/less than monthly/never) in 3 domains: conversation/reading/media. We classified as bilingual participants who reported being able to communicate in L2.

### Cognitive Tests

#### General Fluid-Type Intelligence (g-Factor)

This consisted of a composite of 6 nonverbal tests: Letter–Number Sequencing, Matrix Reasoning, Block Design, Digit Symbol and Symbol Search from the Wechsler Adult Intelligence Scale-III, UK edition (WAIS-III), and Digit Span Backward from the Wechsler Memory Scale-III, UK edition (WMS-III).

#### Memory

This consisted of a composite of Logical Memory (immediate/delayed), Spatial Span (forward/backward), Verbal Paired Associates (immediate/delayed), Digit Span Backward from the WMS-III, and Letter Number Sequencing from the WAIS-III.

#### Speed of Information Processing

This consisted of a composite of Symbol Search and Digit Symbol (WAIS-III), visual inspection time, and simple and choice reaction times.^12^

#### Moray House Test

This is a paper and pencil general cognitive test, including mainly verbal reasoning tasks^13^ (repetition of the test from 1947^10^).

#### Vocabulary/Reading

The National Adult Reading Test (NART)^14^ examined the pronunciation of 50 irregular English words.

#### Verbal Fluency

Participants were asked to say as many words as possible beginning with letters C, F, and L, with a 1-minute time limit for each.

### Data Analysis

As CI is predictive of cognitive functioning in old age,^15^ we adjusted for it when examining the effects of bilingualism on cognitive performance. Outcome variables were Winsorized at the 1st percentile and standardized with zero mean and unit standard deviation. Each was separately modeled as the outcome of multiple linear regression in which the focal predictor was a given variable related to bilingualism, controlling for exact age at testing, sex, and social class (subject's and their father's).

Three bilingualism-related variables, graded into 3 levels, were considered separately: age of acquisition of L2 (never/early/late), number of languages (monolingual/bilingual/multilingual), and the frequency of L2 usage (no second language/no active use/active use). A dummy variable regression model was specified to estimate the effects of bilingualism variables upon the relationship between cognition at age 70 years and CI at age 11 years, adjusted for age at testing, sex, and social class (subject's and their father's). The dummy variables representing levels of bilingualism were coded so as to measure effects relative to a monolingual reference. The model included the main effect of bilingualism and its interaction with CI. We interpreted these effects as additions respectively to the intercept and slope of the predicted relationship between cognition at age 70 years and CI (Fig). Where the interaction with CI (intelligence quotient [IQ] at age 11 years) was significant, we report effects of bilingualism at 3 points along the scale of IQ at age 11 years (mean/5th/95th percentile) by refitting the models with CI centered on these 3 points respectively.

**Figure 1:**
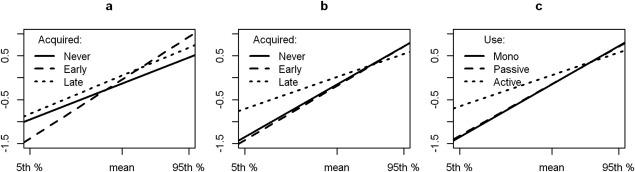
(A–C) Interaction between bilingualism, childhood intelligence quotient (IQ), and cognitive performance at age 73 years. (A) Memory in relation to the age of acquisition of the second language. (B) Moray House Test (MHT) in relation to the age of acquisition of the second language. (C) MHT in relation to the pattern of use of the second language. The abscissa in all 3 graphs is the IQ measured at age 11 years. The ordinate is g-memory (A) and MHT (B, C). (A, B) Never = monolingual group; Early = before age 18 years; Late = after age 18 years. (C) Mono = monolingual; Passive = second language not used in the past 5 years; Active = second language used actively in the past 5 years.

## Results

Two hundred sixty-two participants reported having learned at least 1 language other than English to a degree allowing them to communicate. One hundred ninety-five learned the second language before the age of 18 years (of those, 19 before the age of 11 years), and 65 thereafter. One hundred sixty individuals knew 2, 61 knew 3, 16 knew 4, and 8 knew 5 languages (the last 3 groups were merged into “multilinguals”). One hundred seventy were using only English in their everyday life, whereas 90 used their second language in at least in 1 of the 3 domains.

### Age of Acquisition

Main effects of early acquisition were observed on the g-factor (0.191, *p* = 0.029) and the NART (0.396, *p* < 0.001), and of late acquisition on the g-factor (0.317, *p* < 0.009), processing speed (0.328, *p* = 0.017), and the NART (0.288, *p* < 0.001; Table1).

**TABLE 1:** The Association between Different Types of Bilingualism and Cognitive Ability at Age 73 Years

Outcome Variables	Age of Acquisition				Number of Languages				Frequency of Use			
	Early/Late	Estimate	SE	Pr(>|t|)	2/Multi	Estimate	SE	Pr(>|t|)	Passive/Active	Estimate	SE	Pr(>|t|)
g-Factor		−0.24	0.05			−0.23	0.05			−0.23	0.05	
	Early	0.19	0.09	0.03^a^	2	0.18	0.09	0.06	Passive	0.23	0.08	0.01^a^
	Late	0.28	0.12	0.02^a^	Multi	0.40	0.13	<.01^a^	Active	0.29	0.13	0.03^a^
g-Memory		−0.14	0.06			−0.13	0.06			−0.13	0.06	
	Early	0.09	0.09	0.33	2	0.07	0.10	0.50	Passive	0.13	0.09	0.16
	Late	0.18	0.13	0.14	Multi	0.29	0.16	0.08	Active	0.17	0.14	0.23
g-Speed		−0.14	0.06			−0.14	0.06			−0.14	0.06	
	Early	−0.08	0.10	0.40	2	−0.01	0.11	0.90	Passive	0.01	0.10	0.95
	Late	0.30	0.14	0.03^a^	Multi	0.13	0.15	0.41	Active	0.21	0.15	0.16
MHT		−0.15	0.05			−0.16	0.05			−0.10	0.07	
	Early	−0.03	0.08	0.70	2	0.02	0.08	0.84	Passive	0.01	0.07	0.88
	Late	0.17	0.10	0.11	Multi	0.17	0.12	0.14	Active	0.21	0.12	0.08
NART		−0.17	0.04			−0.17	0.04			−0.16	0.04	
	Early	0.39	0.07	<.01^a^	2	0.34	0.08	<.01^a^	Passive	0.28	0.07	<.01^a^
	Late	0.27	0.10	0.01^a^	Multi	0.58	0.11	<.01^a^	Active	0.58	0.11	<.01^a^
VFT		−0.06	0.06			−0.06	0.06			−0.06	0.06	
	Early	0.16	0.10	0.11	2	0.05	0.11	0.61	Passive	0.19	0.09	0.04^a^
	Late	0.24	0.14	0.08	Multi	0.37	0.15	0.02^a^	Active	0.19	0.15	0.21

The table shows regression estimates for 6 cognitive outcome variables (g-factor, g-memory, g-speed, MHT, NART, VFT) and different types of bilingualism (early vs late acquisition, bi- vs multilingualism, passive vs active). The first row for each outcome is the intercept of the monolingual reference line (see text). The second and third rows show the change in intercept relative to the reference (hence the effect of different types of bilingualism). Where interactions were significant, each marginal main effect represents the outcome change per unit of the covariate with other variables held constant at their respective centered values.

Age of second language acquisition: Early = acquired before age 18 years; Late = acquired after age 18 years. Number of languages acquired: 2 = 2 languages (bilingual); Multi = ≥3 (multilingual). Frequency of use of the second language: Passive = no active use in the past 5 years; Active = active use in the past 5 years.

a Significant effects.

g-Factor = general fluid intelligence factor; g-Memory = memory factor; g-Speed = processing speed factor; MHT = Moray House Test; NART = National Adult Reading Test; SE = standard error; SE = standard error; VFT = Verbal Fluency Test.

A significant association between CI and cognitive performance at age 73 years was found in memory (0.019, *p* < 0.005) for the early acquisition group, and in the Moray House Test (MHT; −0.017, *p* = 0.023) for the late acquisition group (Table2; see Fig). In memory, an effect of early bilingualism was noted in the group with high CI (95th percentile; 0.476, *p* < 0.001). In contrast, on the MHT, the lower CI group benefitted from late bilingualism (5th percentile; 0.662, *p* = 0.010).

**TABLE 2:** Interactions between the Types of Bilingualism and Childhood Intelligence (IQ at Age 11 Years) in the Prediction of Cognitive Performance at Age 73 Years

Bilingual Type	g-Factor	g-Memory	g-Speed	MHT	NART	VFT
**Acquired**						
Early	0.01 (0.10)	0.02 (0.01)^a^	0.01 (0.08)	0.00 (0.8)	−0.01 (0.29)	0.01 (0.26)
Late	−0.01 (0.11)	0.00 (0.83)	−0.01 (0.28)	−0.02 (0.02)^a^	−0.01 (0.18)	−0.01 (0.38)
**Number**						
2	−0.00 (0.82)	0.01 (0.10)	0.00 (0.86)	−0.00 (0.54)	−0.01 (0.16)	0.01 (0.18)
Multi	0.01 (0.31)	0.01 (0.22)	0.01 (0.29)	−0.01 (0.29)	−0.01 (0.08)	0.00 (0.8)
**Usage**						
Passive	0.00 (0.50)	0.01 (0.09)	0.01 (0.38)	0.00 (0.87)	−0.01 (0.29)	0.00 (0.92)
Active	0.00 (0.96)	0.02 (0.08)	−0.01 (0.60)	−0.02 (0.03)^a^	−0.01 (0.09)	0.01 (0.44)

The table shows the estimated interaction effects between IQ at age 11 years and the dummy variables representing the bilingualism-related variables. Each bilingualism variable had 3 levels. The table shows comparisons between 2 of them and the corresponding reference level representing monolingualism. The table shows standardized effects with probability values in parentheses.

a Significant interaction effects. The 3 relevant interactions are illustrated in the Figure.

g-Factor = general fluid intelligence factor; g-Memory = memory factor; g-Speed = processing speed factor; IQ = intelligence quotient; MHT = Moray House Test; NART = National Adult Reading Test; VFT = Verbal Fluency Test.

### Number of Languages

Bilingualism had a main effect on the NART (0.354, *p* < 0.001). Multilingualism had an effect on the g-factor (0.405, *p* = 0.003), the NART (0.592, *p* < 0.001), and verbal fluency (0.371, *p* = 0.016; see Table1). No significant interactions were observed.

### Frequency of Use

Main effects of passive bilingualism were noted on the g-factor (0.244, *p* = 0.004), the NART (0.292, *p* < 0.001), and verbal fluency (0.200, *p* = 0.037; see Table1). Main effects of active bilingualism were found on the g-factor (0.288, *p* = 0.031) and the NART (0.585, *p* < 0.001).

A significant interaction was found between CI and performance at age 73 years for the active bilingual group on the MHT (−0.017, *p* = 0.034; see Fig, C). On this test, a significant effect of active bilingualism occurred only for lower CI (5th percentile; 0.694, *p* = 0.028).

## Discussion

Our results suggest a protective effect of bilingualism against age-related cognitive decline independently of CI. The effects are not explained by other variables, such as gender, socioeconomic status, or immigration. Importantly, we detected no negative effects of bilingualism. The cognitive effects of bilingualism showed a consistent pattern, affecting reading, verbal fluency, and general intelligence to a higher degree than memory, reasoning, and speed of processing. The effect on the NART could be explained by its loanwords with cognates in other languages: bilingualism leads to higher familiarity and hence better performance. The effects on general intelligence are likely to be related to frontal executive advantages, the best documented nonverbal cognitive feature of bilingualism.^8^,^9^

In terms of types of bilingualism, early versus late acquisition showed differential effects, depending on childhood IQ. Overall, individuals with high intelligence seem to benefit more from early acquisition and those with low intelligence from late acquisition, but neither group showed negative effects. Early and late acquisition of a second language might have different effects on frontal executive functions,^16^ possibly modulated by baseline intelligence.

Knowing 3 or more languages produced stronger effects than knowing 2. This variable has yielded contradictory results in previous studies^1^,^4^,^5^ and requires further research. Little difference was found between active and passive bilinguals, possibly due to low frequency of second language use, even in “active bilinguals.” However, it is conceivable that acquisition of a second language leaves lasting cognitive traces independently of its subsequent use. If bilinguals automatically and unconsciously activate both languages,^17^ they constantly need to select, monitor, and suppress linguistic information, stimulating frontal executive functions.^18^–^20^

The observed effect sizes are comparable to those reported for other factors contributing to differences in cognitive ability and cognitive change, such as the effect of variation in the gene for apolipoprotein E, physical fitness, and (not) smoking.^7^ Accordingly, the interpretation of our data should be in terms of cognitive epidemiology, rather than clinical application to an individual. As a small reduction in a population's blood pressure can have a sizeable effect on the number of strokes despite blood pressure accounting for only a small variation in stroke,^21^ a modest change in the proportion of people who learn 1 or more extra languages could have a population effect on cognitive pathology rates.

Our study has limitations. The knowledge of language was defined by a questionnaire, not proficiency. Only few participants acquired their second language before age 11 years, so we could not study the classical cases of parallel, perfect, early acquisition of both languages. However, this limitation is also a strength. Millions of people across the world acquire their second language later in life: in school, university, or work, or through migration or marriage to a member of another linguistic community. Many never reach native-like perfection. For this population, our results are particularly relevant; bilingualism in its broad definition, even if acquired in adulthood, might have beneficial effects on cognition independent of CI.
